# A new class of polyphenolic carbosilane dendrimers binds human serum albumin in a structure-dependent fashion

**DOI:** 10.1038/s41598-024-56509-0

**Published:** 2024-03-11

**Authors:** Marika Grodzicka, Sylwia Michlewska, Adam Buczkowski, Szymon Sekowski, Cornelia E. Pena-Gonzalez, Paula Ortega, Francisco Javier de la Mata, Janusz Blasiak, Maria Bryszewska, Maksim Ionov

**Affiliations:** 1 Department of General Biophysics, Faculty of Biology and Environmental Protection, University of Lodz, Pomorska 141/143, 90-236 Lodz, Poland; 2 Department of General Biophysics, The Bio-Med-Chem Doctoral School of the University of Lodz and Lodz Institutes of the Polish Academy of Sciences, 21/23 Matejki, 90-237 Lodz, Poland; 3 Laboratory of Microscopic Imaging and Specialized Biological Techniques, Faculty of Biology and Environmental Protection, University of Lodz, Banacha 12/16, 90-237 Lodz, Poland; 4 Division of Biophysical Chemistry, Department of Physical Chemistry, Faculty of Chemistry, University of Lodz, Pomorska 165, 90-236 Lodz, Poland; 5 Laboratory of Molecular Biophysics, Department of Microbiology and Biotechnology, Faculty of Biology, University of Bialystok, Ciolkowskiego 1J, 15-245 Bialystok, Poland; 6 Department of Organic and Inorganic Chemistry, and Research Institute in Chemistry “Andrés M. del Río” (IQAR), Spain and Instituto Ramon y Cajal de Investigacion Sanitaria, IRYCIS, Universidad de Alcalá, Colmenar Viejo Road, Km 9, 100, 28034 Madrid, Spain; 7 Networking Research Center On Bioengineering, Biomaterials and Nanomedicine (CIBER-BBN), Madrid, Spain; 8 Faculty of Medicine, Collegium Medicum, Mazovian Academy in Plock, Pl. Dabrowskiego 2, 09-402 Plock, Poland

**Keywords:** Polyphenolic dendrimers, Serum human albumin, Zeta potential, Circular dichroism, Isothermal titration calorimetry, Nanoscience and technology, Nanoparticles

## Abstract

The use of dendrimers as drug and nucleic acid delivery systems requires knowledge of their interactions with objects on their way to the target. In the present work, we investigated the interaction of a new class of carbosilane dendrimers functionalized with polyphenolic and caffeic acid residues with human serum albumin, which is the most abundant blood protein. The addition of dendrimers to albumin solution decreased the zeta potential of albumin/dendrimer complexes as compared to free albumin, increased density of the fibrillary form of albumin, shifted fluorescence spectrum towards longer wavelengths, induced quenching of tryptophan fluorescence, and decreased ellipticity of circular dichroism resulting from a reduction in the albumin α-helix for random coil structural form. Isothermal titration calorimetry showed that, on average, one molecule of albumin was bound by 6–10 molecules of dendrimers. The zeta size confirmed the binding of the dendrimers to albumin. The interaction of dendrimers and albumin depended on the number of caffeic acid residues and polyethylene glycol modifications in the dendrimer structure. In conclusion, carbosilane polyphenolic dendrimers interact with human albumin changing its structure and electrical properties. However, the consequences of such interaction for the efficacy and side effects of these dendrimers as drug/nucleic acid delivery system requires further research.

## Introduction

Carbosilane dendrimers, similarly to their poly(amidoamine) (PAMAM) and poly(propylene imine) (PPI) counterparts, are hyperbranched and monodispersed macromolecules explored as drug and nucleic acid carriers in the therapy of many diseases, including cancer and viral infections^1^. Therefore, two aspects of dendrimers as drug/gene carriers must be considered: their efficacy as a carrier and their toxicity. The structure of dendrimers may be modified to increase the former and decrease the latter. The presence of multivalent functional end groups in the dendrimer structure creates ample opportunities for their modification aimed at acquiring specific functions, including the ability to modify the properties of biological macromolecules. Such modifications may result in increased efficacy of the carried drug, which is the case in anticancer therapy when a modified dendrimer has anti-cancer properties^2,3^.

Functionalization of dendrimers with polyphenolic residues can give or improve their antioxidant, anti-radical, antiviral, and anti-bacterial properties^4^. The functionalization of carbosilane dendrimers with caffeic acids increased their antioxidant and anti-radical activity^5^. Despite many advantages of positively charged dendrimers, they can form microholes in biological membranes, that may underlie their cellular and organismal toxicity^2^. The incorporation of polyethylene glycol (PEG) residues into the dendrimer structure was reported to have beneficial consequences, increasing its bioavailability and decreasing its toxicity^2^. In addition, PEG is non-immunogenic, water-soluble, nontoxic and can reduce interactions of dendrimers with serum proteins^7,8^.

Drug and nucleic acid carriers encounter many objects on their way to the target site. Albumin is the most abundant protein in blood and therefore its interaction with drug and gene carriers should be considered as it may lead to toxicity, change bioavailability, and potential therapeutic effect. Human serum albumin (HSA) is a monomeric protein with one tryptophan residue and three homologous domains. Each domain has 2 subdomains to which different molecules can be attached. Subdomain IIA which contains the only HSA tryptophan residue is the main hydrophobic site for ligand binding and dendrimers interact with this site mainly through hydrophobic interactions and may form “protein corona”^9^. This effect is crucial for the medical application of dendrimers because their absorption on the albumin surface can change their properties, bioavailability, and distribution^10^. In the present work, the interaction of a new class of 1^st^ generation carbosilane dendrimers functionalized with various numbers of caffeic acids and PEG residues with human serum albumin in vitro was investigated with a plethora of biochemical and biophysical techniques.

## Materials and methods

### Dendrimers

**Figure 1:**
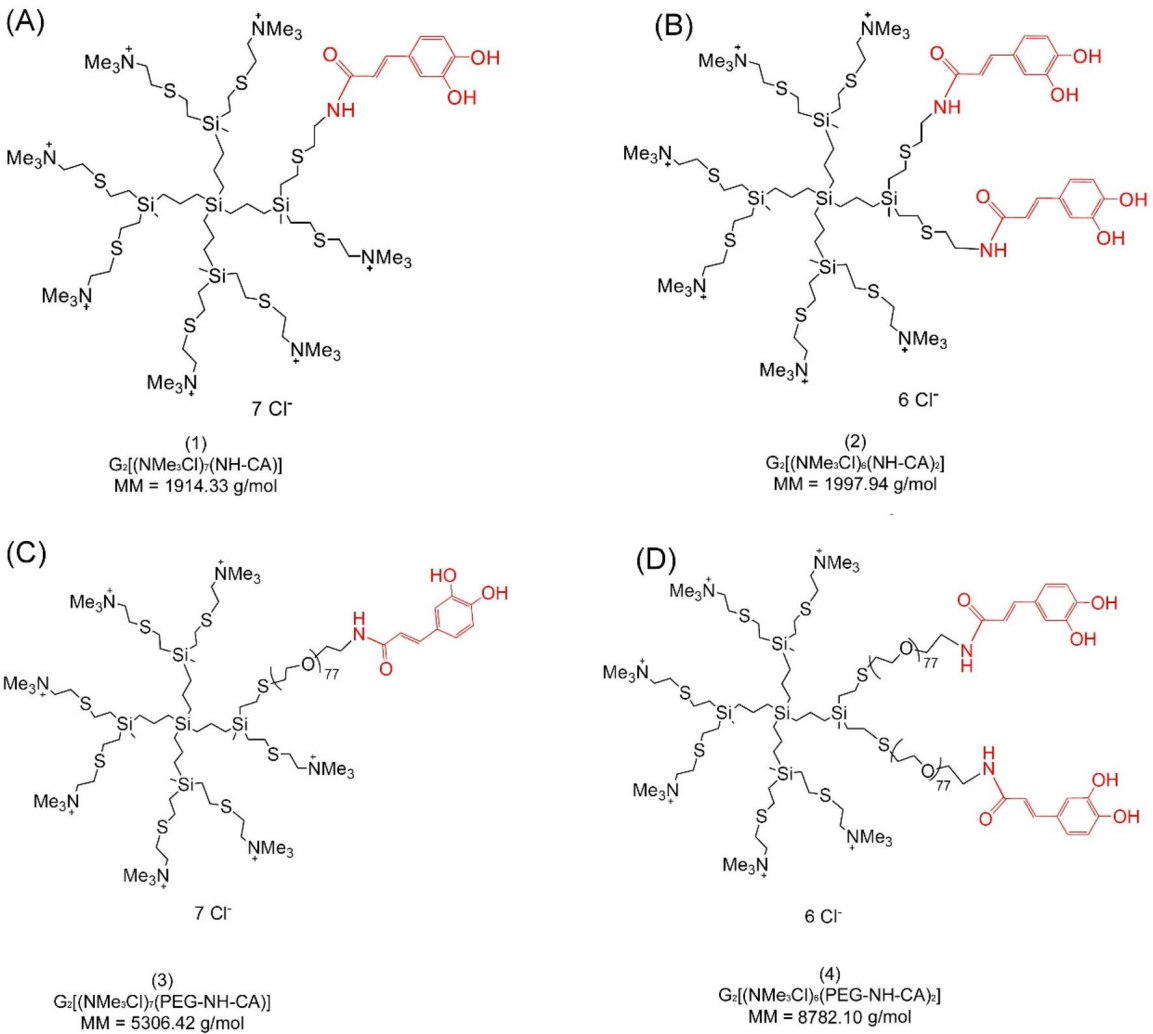
Structure, chemical formula, and molar mass (MM) of polyphenolic carbosilane dendrimers with ammonium surface groups functionalized with caffeic acid and polyethylene glycol (PEG). (**A**) compound (**1**), G_2_[(NMe_3_Cl)_7_(NH-CA)]; (**B**) compound (**2**), G_2_[(NMe_3_Cl)_6_(NH-CA)_2_]; (**C**) compound (**3**), G_2_[(NMe_3_Cl)_7_(PEG-NH-CA)]; (**D**) compound (**4**), G_2_[(NMe_3_Cl)_6_(PEG-NH-CA)_2_]. Caffeic acid moieties are marked in red.

Four water-soluble 1st generation heterofunctionalized polyphenolic carbosilane dendrimers G_2_[(NMe_3_Cl)_7_(NH-CA)] (**1**); G_2_[(NMe_3_Cl)_6_(NH-CA)_2_] (**2**); G_2_[(NMe_3_Cl)_7_(PEG-NH-CA)] (**3**) and G_2_[(NMe_3_Cl)_6_(PEG-NH-CA)_2_] (**4**) with ammonium and caffeic acid surface groups were used in this study. The structure and molar mass of dendrimers are presented in Fig. 1. The procedure of the synthesis of these compounds was described elsewhere^5^.

### Zeta potential and zeta size

The hydrodynamic diameter and the zeta potential of human serum albumin in the presence of the dendrimers were measured using a Zetasizer Nano-ZS photon correlation spectrometer (Malvern Instruments, Malvern, Worcestershire, UK). Measurements were performed in a 10 mmol/L phosphate buffer at pH 7.4 at 25 °C. Zeta potential values were calculated directly from the Helmholtz-Smoluchowski equation and Malvern Zetasizer Nano software v3.30 (Malvern Panalytical Ltd., Malvern, UK) was used for data analysis. At least 3 separate replicates were performed for each experiment.

### Transmission electron microscopy

The ultrastructure of the albumin-dendrimer complexes at the molar ratio 1:10 was visualised using a JEOL-1010 (JEOL, Tokyo, Japan) transmission electron microscope. The samples in 10 mmol/L Na-phosphate buffer, pH 7.4, were placed on 200 mesh copper grids with a carbon surface (Ted Pella, Inc, Redding, CA, USA). Samples were stained with 2% uranyl acetate for 2 min, washed with deionized water, and dried at room temperature. Images were taken at a magnification of 100,000 × and analysed with conventional software.

### Fluorescence spectroscopy

To analyse the character of interaction between albumin and dendrimers, tryptophan fluorescence was measured using a Perkin-Elmer LS-55B fluorescence spectrometer. Appropriate amounts of dendrimers were added to albumin solution (4 µmol/L) with the final albumin:dendrimer molar ratios of 1:1, 1:2, 1:4, 1:6, 1:8 and 1:10. The excitation wavelength for tryptophan was λ_exc_ = 295 nm and fluorescence spectra were taken in the 305–450 nm range. Excitation and emission slits were set at 2.5 and 8.0 nm, respectively. Fluorescence spectra of albumin were corrected for the proper baselines. All measurements were performed at 25 °C in 3 independent replicates. Stern–Volmer constant (*K*_SV_), quenching constant (*k*_q_), and binding constant (logK_b_) were calculated.

### Circular dichroism

The circular dichroism (CD) spectra of dendrimer/HSA complexes were measured using a J-815 CD spectrometer (Jasco, Tokyo, Japan). The concentration of albumin was 0.25 µmol/L. Protein/dendrimer complexes were prepared in 10 mmol/L phosphate buffer, pH 7.4, at molar ratios ranging from 1:1 to 1:10. Measurements were made at room temperature, in 5-mm path-length quartz cuvettes. The recording parameters were scan speed 50 nm/min, step resolution 1 nm, response time 4 s, bandwidth − 1.0 nm, slit—auto. CD spectra were obtained in the wavelength range 195–260 nm, as the average of a minimum of 3 independent experiments composed from 3 accumulated scans of each replication. The protein secondary structure and distribution of α-structure and β-sheet percentage under the influence of dendrimers were calculated using the CDNN software available at: https://cdnn-circular-dichroism-spectroscopy-deconvolution.updatestar.com.

### Isothermal titration calorimetry

An isothermal titration calorimetric study was performed to measure the thermodynamic properties of the interaction between the dendrimers and albumin. Albumin solution (182 μl; 100 μmol/L in a cell) was titrated by adding 40 × 5 μl doses of a 2 mmol/L dendrimer solution in 5-min intervals. Measurements of the thermal effects of the titration were carried out at 25 °C with a stirring rate of 125 RPM in auto-equilibrate mode. During titration, the molar ratio of dendrimer to albumin increased from 1.1:1 to 40:1. The thermal effects of the direct interaction of albumin with dendrimers were calculated by subtracting the effects of dilution of the dendrimer from the corresponding thermal effects of the titration of albumin solution with the dendrimer solution. The binding isotherms were analysed in NanoAnalyze software by a non-linear multiparameter regression using the independent-site model to calculate the stoichiometric parameter $n$, equilibrium binding constant $K$, and standard thermodynamic functions of the process of albumin binding with dendrimers: enthalpy $\Delta H$, entropy, $\Delta S$ and Gibbs free energy $\Delta G$.

### Data analysis

All results were obtained from at least 3 independent experiments and are presented as mean ± SD (standard deviation). Statistical analyses were performed for paired samples using GraphPad Prism version 8.0.1 software (GraphPad Software, Boston, MA, USA). The normality of the study sample was checked using the Shapiro–Wilk test. In the case of a normal distribution of data, the Student's t-test was used, while in other cases the ANOVA post hoc Dunnett's test was used. The 95% confidence interval was set as the confidence interval.

## Results

### Dendrimers form complexes with albumin as revealed by the zeta potential, zeta size, and transmission electron microscopy

**Figure 2:**
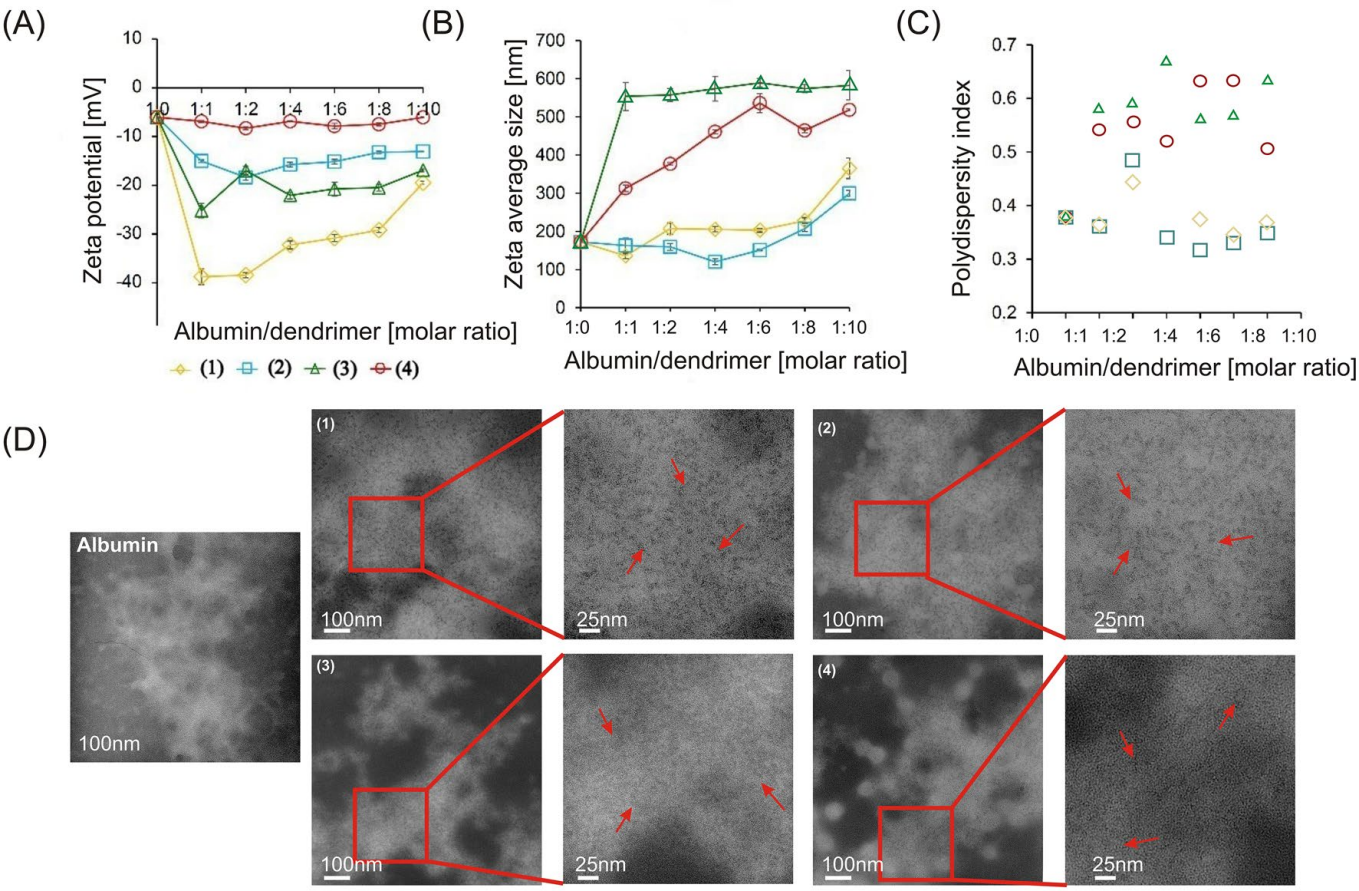
Dose-dependent effects of polyphenolic dendrimers on zeta potential (**A**), zeta size (**B**), and polydispersity index (**C**). Data points represent mean ± SD obtained from a minimum 3 experiments and each experiment was done in 7 replicates for dendrimer (**1**) (yellow line open diamonds), (**2**) (blue line, open squares) (**3**) (green line, open triangles) and (**4**) (red line, and open circles). Panels (**D**) represent the ultrastructure of human serum albumin in the presence of dendrimers. Bars 100 nm and 25 nm, to obtain greater contrast, the color of the micro images has been inverted.

The zeta average size of free albumin was 172.15 ± 15.54 nm and the addition of dendrimers led to an increase in particle size indicating the binding of dendrimer to albumin (Fig. 2B). The highest hydrodynamic diameters of the dendrimer-albumin complexes were observed in the presence of the dendrimers (**3**) and (**4**). In that case, the size of the complexes at the highest tested molar ratio 1:10 increased to 582.45 ± 38.51 nm (**3**) and 518.33 ± 2.13 nm (**4**). The size of complexes formed with dendrimers (**1**) and (**2**) were smaller, 356.26 ± 26.89 and 299 ± 7.37 respectively, as they did not have any polyethylene glycol chain. The polydispersity index (PDI) of the complexes formed by compounds (**1**) and (**2**) was around 0.4, while in the presence of compounds (**3**) and (**4**) it increased to 0.6 and higher values (Fig. 2C).

The zeta potential of free albumin was − 6.04 ± 0.39 mV and the addition of dendrimers (**1**), (**2**), and (**3**) decreased it. This effect was most pronounced with dendrimers with one caffeic acid residue. Zeta potential values with dendrimers (**1**) and (**3**) were − 38.76 ± 0.62 mV and − 25.17 ± 0.83 mV, respectively, while for (**3**) it was − 18.36 ± 0.28 mV and for (**4**) − 8.30 ± 0.22 mV (Fig. 2A).

Free albumin structure presented a fibrillary form, and the presence of dendrimers made albumin molecules slightly more compact (Fig. 2D). This effect was maximal for the complexes formed by dendrimer (**2**) and minimal for (**4**). All complexes were presented as fibrillar structures with small globular structures (Fig. 2D -arrows).

### Fluorescence quenching confirms the formation of albumin-dendrimer complexes

**Figure 3:**
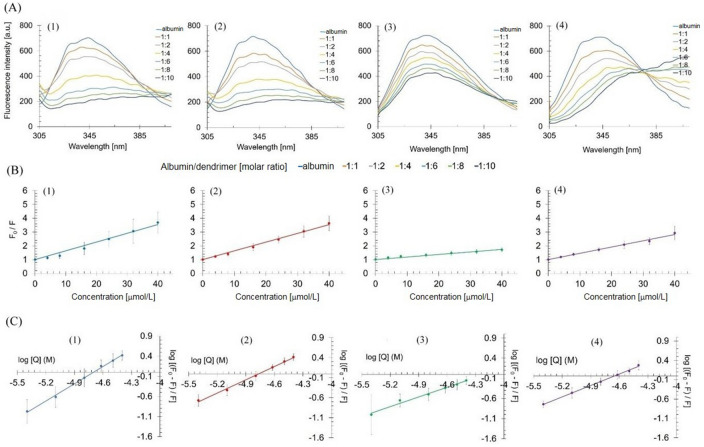
Fluorescence emission spectra of albumin-dendrimer complexes for dendrimers (**1–4**) at the albumin/dendrimer molar ratios 1–10 (**A**). Stern–Volmer plots of tryptophan fluorescence quenching in the presence of increasing concentrations of dendrimers (**B**), double-logarithmic plot of tryptophan fluorescence quenching at albumin concentration 4 µmol/L (**C**). Data points represent mean ± SD obtained from a minimum 3 separate experiments.

The binding of dendrimers changed the fluorescence spectrum of the tryptophan residue in albumin towards longer wavelengths (Fig. 3). The most pronounced effect was observed for the dendrimer (**4**).

To quantitatively assess the effect of dendrimers on the fluorescence properties of albumin, we calculated the Stern–Volmer constant (*K*_SV_), which indicates the efficacy of dendrimers in quenching fluorescence of tryptophan (Table 1). We also calculated the quenching constant *k*_q_ to differentiate between the static and dynamic character of quenching by dendrimers and the logarithm of binding constant (log*K*_b_) for all dendrimers (Table 1)^4^. Since *k*_q_ for each dendrimer was greater than 2 × 10^10^ M^−1^ s^−1^, we concluded that the dendrimers quenched fluorescence via the static mechanism by the formation of complexes with albumin.

**Table 1:** Protein-polyphenolic dendrimers interaction parameters calculated based on fluorescence results.

Compound	*K*_*SV*_ [M^−1^]	*k*_*q*_ [M^−1^ s^−1^]	*logK*_*b*_
**(1)**	(6.35 ± 2.26) × 10^4^	(1.27 ± 0.45) × 10^13^	6.72 ± 0.62
**(2)**	(6.34 ± 1.27) × 10^4^	(1.27 ± 0.25) × 10^13^	5.26 ± 0.20
**(3)**	(1.86 ± 0.49) × 10^4^	(0.37 ± 0.10) × 10^13^	3.38 ± 1.39
**(4)**	(4.54 ± 1.05) × 10^4^	(0.91 ± 0.21) × 10^13^	4.57 ± 0.29

The most pronounced quenching effect was observed for dendrimers (**1**) and (**2**), which were characterized by the highest K_SV_. Consequently, these dendrimers were characterized by the highest log*K*_b_.

### Dendrimers slightly change the secondary structure of albumin

**Figure 4:**
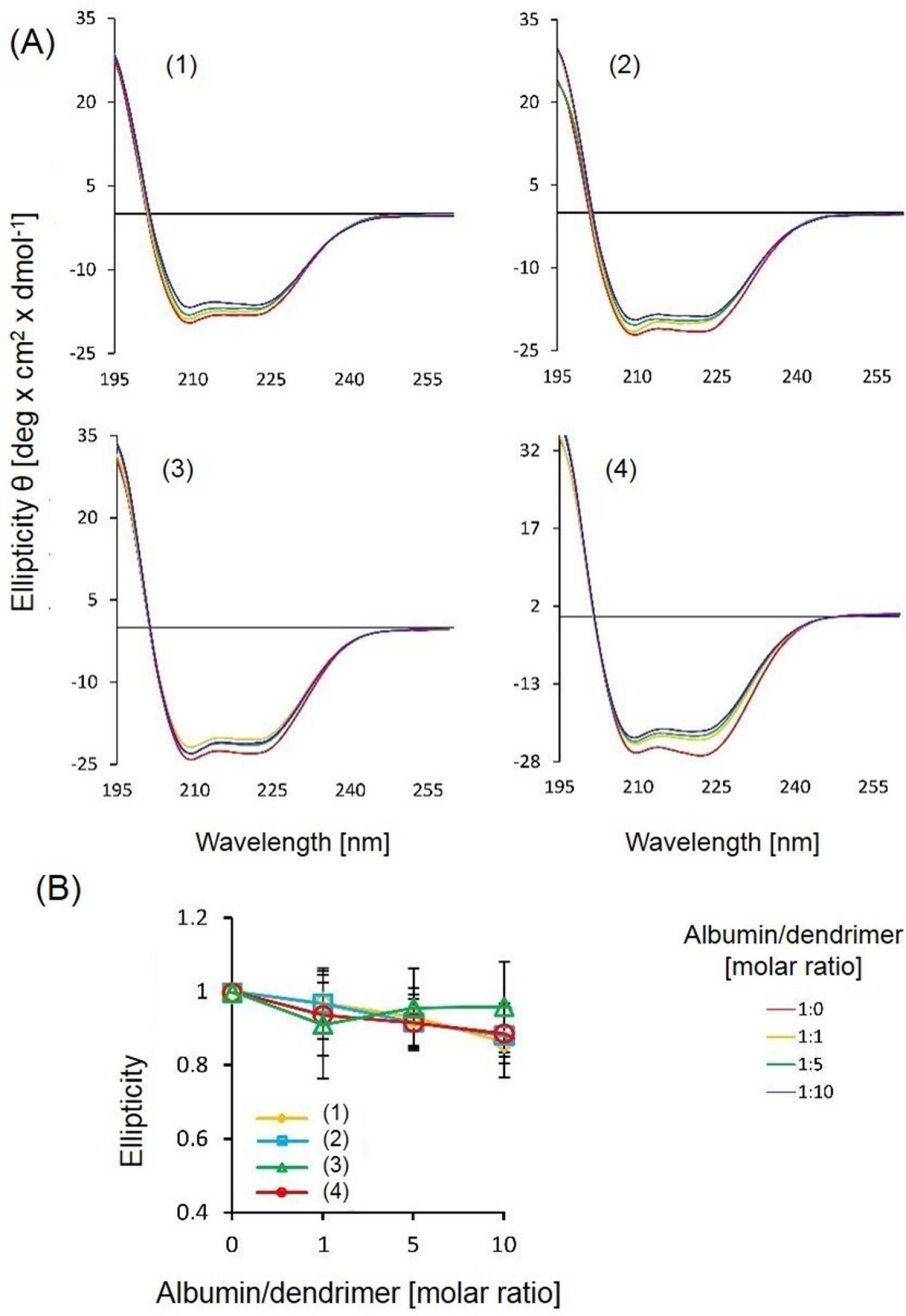
Circular dichroism spectra of free albumin and its complexes with the dendrimers (**A**). Relative ellipticity of the albumin-dendrimer complexes at its different ratios. Data are presented as means ± SD obtained from a minimum of 3 separate experiments.

We observed two standard negative CD bands for albumin at 208 and 222 nm, which were slightly changed in the presence of the dendrimers (Fig. 4). The changes in the secondary structure of albumin induced by the dendrimers were evaluated with the CDNN software (Table 2). Slight conformational changes in the albumin secondary structure were observed in the presence of the dendrimers.

**Table 2:** Contribution of α-helix, β-sheet, and random coil to the overall structure of free albumin and albumin-dendrimer complexes at 1:10 molar ratio calculated with CDNN Software.

Dendrimer	α-helix (*%*)	β-sheet (*%*)	Random coil (*%*)
Free albumin	56.73 ± 2.51	13.26 ± 0.35	17.47 ± 1.28
**(1)**	53.40 ± 5.65	13.63 ± 0.85	21.07 ± 1.77
**(2)**	55.53 ± 7.59	13.36 ± 1.09	19.60 ± 3.32
**(3)**	57.00 ± 1.83	13.23 ± 0.28	18.23 ± 0.67
**(4)**	57.45 ± 10.1	13.15 ± 1.48	18.60 ± 4.24

### On average, one molecule of albumin is bound by 6–10 molecules of dendrimers

**Figure 5:**
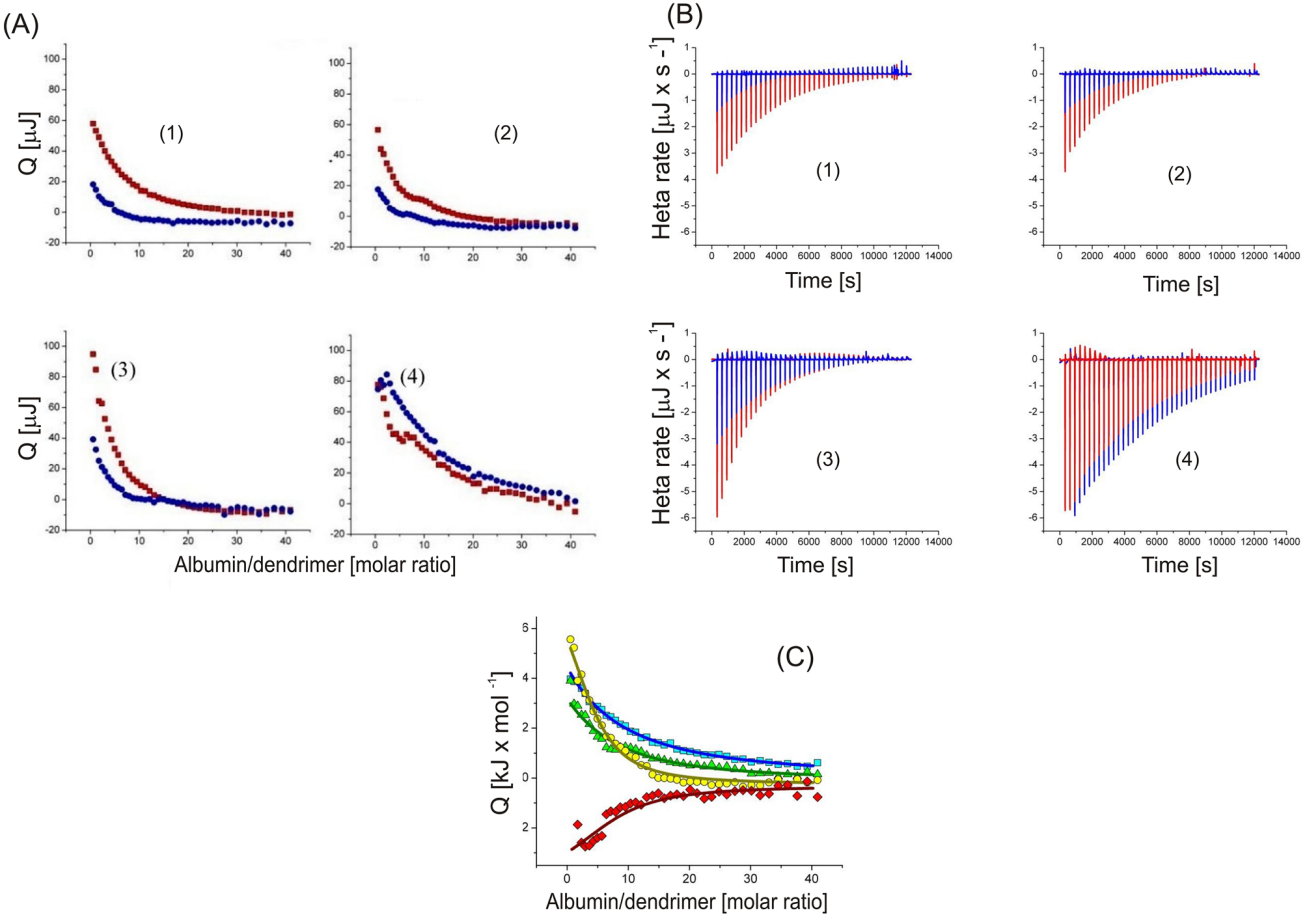
Integrated thermal effects of the isothermal titration calorimetry titration of 100 µmol/L HSA solution with 2 mmol/L solution of each dendrimer (red squares) and corresponding effects of the dilution of dendrimer (blue circles) (**A**). Curves of thermal power as a function of time during titration of 100 µmol/L albumin solution with 2 mmol/L dendrimer solution (red peaks) and dilution of 2 mmol/L dendrimer solution into buffer without albumin (blue peaks). Downward peaks correspond to endothermic heat effects (**B**). Thermal effects of direct interactions (per mole of injectant) between albumin and polyphenolic carbosilane dendrimers as a function of dendrimer to albumin mole ratio. All titrations were carried out at 25 °C in aqueous 10 mmol/L phosphorous buffer solution pH 7.4.

Isothermal titration calorimetry analysis (Fig. 5) enabled to calculation some parameters characterizing complexes formed by albumin and dendrimers, namely the stoichiometric parameter n, equilibrium binding constant K, change in enthalpy ∆H, entropy ∆S, and Gibbs free energy ∆G (Table 3).

**Table 3:** Stoichiometric parameter *n*, equilibrium binding constant *K*, change in enthalpy *∆H*, entropy *∆S,* and Gibbs free energy *∆G* of the formation of albumin-dendrimer complexes evaluated by isothermal titration calorimetry.

Dendrimer	$n$	$logK$	$\Delta H$ (kJ mol^–1^)	$T\Delta S$(kJ mol^–1^)	$\Delta G$(kJ mol^–1^)
**(1)**	10 ± 3	3.49 ± 0.57	25 ± 14	45 ± 3	-20 ± 17
**(2)**	9 ± 4	3.81 ± 0.47	15 ± 8	37 ± 9	-22 ± 17
**(3)**	6 ± 4	3.61 ± 0.94	8.3 ± 6.8	29 ± 13	-21 ± 20
**(4)**	7 ± 5	3.76 ± 0.46	-4.0 ± 3.2	17 ± 14	-21 ± 17

These results show that one albumin molecule can be associated with approximately 10 molecules of dendrimers. The binding equilibrium constant $K$ and standard thermodynamic functions indicate that the process of dendrimers binding with albumin was thermodynamically spontaneous ($\Delta G<0$). The driving force behind albumin binding with (**1**), (**2**), and (**3**) dendrimer was a favourable increasing disorder of reagents ($T\Delta S>0$), which overcame the unfavourable endothermic effects of the direct interactions of the reagent ($\Delta H>0$). Interactions of HSA with dendrimer (**4**), with 2 caffeic moieties and 2 PEG chains, were favourable, as both the exothermic enthalpy of binding ($\Delta H<0$) and increasing disorder of reagents ($T\Delta S>0$), reflecting the role of the 2 polar side PEG chains in the structure.

## Discussion

The interaction of dendrimers with serum proteins affects their bioavailability. That interaction may be underlined by various mechanisms, first of all of electrostatic nature, and may not only limit the bioavailability of dendrimers but also underlie their toxicity. In this work, we investigated the interaction of a new class of 1st generation heterofunctional polyphenolic dendrimers modified with caffeic acid with human serum albumin, the most abundant blood protein. We showed that these dendrimers bound albumin in a stochiometric ratio of 5–10 molecules of dendrimers per single molecule of albumin. This binding was confirmed by several methods. Despite the binding, the secondary structure of albumin was not significantly affected.

Dendrimers can bind albumin via electrostatic interactions, hydrogen bonding, hydrophobic interactions, and specific interactions between dendrimer groups and aliphatic acid binding sites of the protein^11–13^. In this study, all dendrimers changed the zeta potential of albumin. Cationic and neutral dendrimers were reported to change the zeta potential of proteins^9^. We showed that the dendrimers (**1**) and (**3**) that were not modified with PEG strongly changed the zeta potential as their inner positive charge was not shielded by PEG as in dendrimers (**3**) and (**4**).

We observed a red-shift effect in our fluorescence experiments, which was the most pronounced for the dendrimer (**4**) that had 2 caffeic acid moieties and PEG. Therefore, that dendrimer might interact with a hydrophilic region of albumin. The formation of complexes of dendrimers and albumin was confirmed in quenching experiments, in which quenching constants were larger than 2 × 10^10^ M^−1^ s^−1^ for all dendrimers. The Stern–Volmer analysis revealed that the dendrimers (**1**) and (**2**), had the largest K_SV_ values, and that corresponded with the magnitude of the quenching effect induced by these compounds. The presence of PEG in the dendrimer structure causes charge masking and an increase in surface density. Therefore, there is a reduced availability of the inner positive charges in any interactions with dendrimers (**3**) and (**4**). On the contrary, the attachment of the caffeic acid residue by the addition of a PEG molecule may result in an easier detachment of this residue from the carbosilane skeleton. Therefore, it is likely that the presence of two PEG molecules results in an easier release of caffeic acid residues, and therefore a higher availability to the carbosilane skeleton.

Our CD study showed that the dendrimers did not change the secondary structure of albumin and observed slight changes in the proportion of α-helix, β-sheet, and random coil in the overall structure of albumin were non-significant. Similar effects were described for cationic PAMAM g3 and g4 dendrimers modified by polyethylene glycol (PEG), and sugar-persubstituted PAMAM dendrimers of the 3rd and 5th generations did not change the secondary structure of albumin^14^. The size and flexibility of dendrimers are crucial in their interaction with proteins, as rigid nanoparticles can change protein structure from α-helix to β-sheets, more flexible particles can rather change the protein structure from α helix to random coil^15^. The weak effect of polyphenolic dendrimers on the albumin secondary structure can be explained by the fact that less hydrophobic dendrimers would exert a smaller effect on the hydrophobic amino acids in the protein composition. While the changes in the percentage of α-helices in the presence of dendrimers provided information about weak changes in the overall protein secondary structure, Trp fluorescence quenching reflected the local changes in the vicinity of the tryptophan residue. This may underline apparent discrepancies in the results we obtained. A similar apparent inconsistency in these 2 methods in the study of dendrimer interactions with thrombin has also been observed^15^. The same studies also showed that the presence of PEG reduced interactions with that serum protein. While dendrimers with reactive aldehyde terminal groups quenched albumin fluorescence and changed the secondary structure of albumin, modification of the terminal groups with phosphonate or PEG quenched Trp fluorescence, but they did not change the conformation of the protein^13^. A similar situation was observed for interactions of other dendrimers with other proteins^16^. 

Isothermal titration calorimetry analysis indicated that 6–10 molecules of polyphenolic dendrimers might associated with one molecule of albumin. This is in line with our previous study showing that a single molecule of HSA could bind on average 6 particles of dendrimers^17^. The differences between the logarithm of K_b_ received from fluorescence quenching and ITC analyses can be explained, similarly to CD, by the difference in local (Trp fluorescence) and global (affinity to the whole of albumin) interactions of dendrimers.

As we stated in the introductory section, dendrimers might interact with HSA through its main hydrophobic site encompassing the tryptophan residue, but the precise nature of this interaction remains unknown. However, some dendrimers can adopt a directional three-dimensional structure exposing their hydrophobic inner core and enabling hydrophobic interactions with suitable domains of a protein^18^.

On of the limitations of our work is associated with the actual structure of the dendrimers. The process of the functionalization of the dendrimers with polyphenolic moieties is stochastic, excluding the determination of the exact position of polyphenolic groups in the dendritic structure. Therefore, the layout of the branches functionalized with polyphenolic residues cannot be unequivocally determined, potentially affecting their interaction with HSA. The question remains whether such affected interaction was of biological significance and whether it was measurable with the techniques that were applied in this work. Further studies with a more controlled procedure of dendrimer synthesis and functionalization may shed light on these problems.

## Conclusions

Carbosilane polyphenolic dendrimers can interact with human serum albumin and this interaction depends on the presence of polyethylene glycol and caffeic acid residues. Therefore, albumin may decrease the bioavailability of these dendrimers, which should be considered in strategies for the use of these dendrimers as drug/gene carriers. Furthermore, the interaction of these dendrimers with albumin may lead to their toxicity and require further research.

## Data Availability

Data are available upon reasonable request from corresponding authors: Sylwia Michlewska Tel.: + 48 42 635 44 31, e-mail: sylwia.michlewska@biol.uni.lodz.pl. Maksim Ionov Tel.: + 48 42 635 43 80, e-mail: maksim.ionov@biol.uni.lodz.pl.

## References

[1] Sepúlveda-Crespo D, Gómez R, De La Mata FJ, Jiménez JL, Muñoz-Fernández M (2015). Polyanionic carbosilane dendrimer-conjugated antiviral drugs as efficient microbicides: Recent trends and developments in HIV treatment/therapy. Nanomedicine.

[2] Michlewska S (2023). Lipid-coated ruthenium dendrimer conjugated with doxorubicin in anti-cancer drug delivery: Introducing protocols. Colloids Surf. B Biointerfaces.

[3] Michlewska S (2023). Ruthenium metallodendrimer against triple-negative breast cancer in mice. Nanomed. Nanotechnol. Biol. Med..

[4] Sanz Del Olmo N (2020). Antioxidant and antibacterial properties of carbosilane dendrimers functionalized with polyphenolic moieties. Pharmaceutics.

[5] Grodzicka M (2022). Heterofunctionalized polyphenolic dendrimers decorated with caffeic acid: Synthesis, characterization and antioxidant activity. Sustain. Mater. Technol..

[6] Somani S (2018). PEGylation of polypropylenimine dendrimers: effects on cytotoxicity, DNA condensation, gene delivery and expression in cancer cells. Sci. Rep..

[7] Jain K, Kesharwani P, Gupta U, Jain NK (2010). Dendrimer toxicity: Let's meet the challenge. Int. J. Pharm..

[8] Thakur S, Kesharwani P, Tekade R, Jain N (2015). Impact of pegylation on biopharmaceutical properties of dendrimers. Polymer.

[9] Shcharbin D (2015). Nanoparticle corona for proteins: Mechanisms of interaction between dendrimers and proteins. Colloids Surf. B Biointerfaces.

[10] Kubczak M (2023). The effect of novel tyrosine-modified polyethyleneimines on human albumin structure—Thermodynamic and spectroscopic study. Colloids Surf. B Biointerfaces.

[11] Klajnert B, Bryszewska M (2002). Fluorescence studies on PAMAM dendrimers interactions with bovine serum albumin. Bioelectrochemistry.

[12] Klajnert B, Stanisławska L, Bryszewska M, Pałecz B (2003). Interactions between PAMAM dendrimers and bovine serum albumin. Biochim. Biophys. Acta.

[13] Moreno S (2015). Synthesis, characterization and biological properties of new hybrid carbosilane–viologen–phosphorus dendrimers. RSC Adv..

[14] Froehlich E, Mandeville JS, Jennings CJ, Sedaghat-Herati R, Tajmir-Riahi HA (2009). Dendrimers bind human serum albumin. J. Phys. Chem. B.

[15] Shcharbin D (2017). Binding of poly(amidoamine), carbosilane, phosphorus and hybrid dendrimers to thrombin-Constants and mechanisms. Colloids Surf. B Biointerfaces.

[16] Ionov M (2016). Effect of dendrimers on selected enzymes–Evaluation of nano carriers. Int. J. Pharm..

[17] Sekowski S, Buczkowski A, Palecz B, Gabryelak T (2011). Interaction of polyamidoamine (PAMAM) succinamic acid dendrimers generation 4 with human serum albumin. Spectrochim. Acta Part A Mol. Biomol. Spectrosc..

[18] Caminade AM (2015). The key role of the scaffold on the efficiency of dendrimer nanodrugs. Nat. Commun..

